# Delay for First Consultation and Its Associated Factors among New Pulmonary Tuberculosis Patients of Central Nepal

**DOI:** 10.1155/2016/4583871

**Published:** 2016-04-10

**Authors:** Wongsa Laohasiriwong, Roshan Kumar Mahato, Rajendra Koju, Kriangsak Vaeteewootacharn

**Affiliations:** ^1^Board Committee of Research and Training Centre for Enhancing Quality of Life of Working Age People (REQW), Khon Kaen University, Khon Kaen, Thailand; ^2^Faculty of Public Health, Khon Kaen University, Khon Kaen, Thailand; ^3^Departments of Internal Medicine, Dhulikhel Hospital, Kathmandu University Hospital, Dhulikhel, Nepal

## Abstract

Tuberculosis (TB) is still a major public health challenge in Nepal and worldwide. Most transmissions occur between the onset of symptoms and the consultation with formal health care centers. This study aimed to determine the duration of delay for the first consultation and its associated factors with unacceptable delay among the new sputum pulmonary tuberculosis cases in the central development region of Nepal. An analytical cross-sectional study was conducted in the central development region of Nepal between January and May 2015. New pulmonary sputum positive tuberculosis patients were interviewed by using a structured questionnaire and their medical records were reviewed. Among a total of 374 patients, the magnitude of patient delay was 53.21% (95% CI: 48.12–58.28%) with a median delay of 32 days and an interquartile range of 11–70 days. The factors associated with unacceptable patient delay (duration ≥ 30 days) were residence in the rural area (adj. OR = 3.10, 95% CI: 1.10–8.72; *p* value = 0.032) and DOTS center located more than 5 km away from their residences (adj. OR = 5.53, 95% CI: 2.18–13.99; *p* value < 0.001). Unemployed patients were more likely to have patient delay (adj. OR = 7.79, 95% CI: 1.64–37.00; *p* value = 0.010) when controlled for other variables.

## 1. Background

TB ranks as the second leading cause of death from an infectious disease after the Human Immunodeficiency Virus (HIV) worldwide (126 cases per a population of 100000 individuals) [1, 2]. In the South Asia region alone, it accounts for 39% of total global burden of TB [3]. Nepal is a landlocked country between India and China with one-fourth of the total population of 26.6 million under the poverty line and 83% living in the rural areas. TB is one of the major public health challenges in Nepal [4–6]. About 45% of the total population are infected, among which 60% are adults. Every year, 45,000 people are estimated to develop active TB, 50% of which develops into infectious pulmonary disease and is the main cause for spreading the disease [7].

Complete treatment consists of a 6-month drug regimen resulting in a negative sputum smear [8]. If diagnosed between 2 and 3 weeks after the onset of clinical symptoms, it is considered as early diagnosis, while a diagnosis beyond 4 weeks of onset is considered as delayed diagnosis [9]. However, incomplete treatment regimens of tuberculosis patients are as high as 20%–50% in both high and low income countries [10, 11].

Delayed diagnosis or incomplete treatment results in prolonged infectiousness, drug resistance, relapse, and even death [10, 12–14]. Patients with undiagnosed pulmonary TB primarily act as reservoirs for transmission. The contagion parameter suggests that, where TB is endemic, each infectious case will result in 20–28 secondary infections [11]. The related literature has revealed that patients' first presentation to health facilities after more than 30 days is associated with geographical condition, health service, age, poverty, sex, alcoholism, substance abuse, history of immigration, educational status, awareness of TB, social beliefs, self-treatment, and stigma [2, 12, 13, 15–17]. Thus, there are many factors that need to be addressed in order to achieve the goal of reducing the TB burden. These types of studies have not been conducted in the study area. Therefore, this study aimed to investigate the duration of delay for the first consultation and associated factors among the new sputum pulmonary tuberculosis cases in central Nepal.

## 2. Materials and Methods

### 2.1. Study Design and Period

An analytical cross-sectional study was conducted on new pulmonary sputum positive pulmonary tuberculosis patients at DOTS centers from January to May 2015.

### 2.2. Study Setting

The study was conducted in central development region (CDR) of Nepal which is one of the five development regions of Nepal that spans all three ecological regions: mountain, hilly, and terai (plain landscape south of the outer foothills of the Himalaya). Headquartered in Hetauda (Makwanpur district), the CDR comprises three administrative zones (Bagmati, Narayani and Janakpur) and 19 districts. The population density in the CDR is 293 inhabitants per square kilometre, which is the highest among all five development regions and significantly above the national average of 157 inhabitants per square kilometre. The CDR consists of 36.81% of the total population of Nepal. The Human Development Index (HDI) of the CDR (0.531) is higher than the national average of 0.509 [4, 5]. This region constitutes 366 TB treatment centers, 1008 treatment subcenters, 4 drug resistant (DR) treatment centers, 37 DR treatment subcenters, and 198 microscopy centers [18].

This region was selected randomly from the five developmental regions. We then randomly selected 5 districts, one from each ecological region. Finally, the data were collected from treatment centers of each selected district by using systematic random sampling.

### 2.3. Sample Size and Sampling Procedure

The sample size of 374 was determined by using the formula for multiple logistic regression (*n* = [*P*(1 − *P*)(*Z*1 − *α* + *Z*1 − *β*)^2^/*B*(1 − *B*)(*P*0 − *P*1)^2^ *∗*1/(1 − *P*)2]) [19]. The proportion was obtained from a previous study in Uganda [20] where *P*  (69%) is proportion of delay in diagnosis, *P*0  (97%) is the proportion of delay in patients who had visited the private facilities, *P*1  (60%) is the proportion of delay in patients who had visited the public facilities, *B* (75%) is proportion of perceiving visiting to the public facilities, *α* = 5%, and 1 − *β* = 84%. New smear positive pulmonary tuberculosis cases above 15 years of age were included in this study, while negative smear, relapse, retreatment, return after defaulter, and patients who had history of prior TB were excluded.

### 2.4. Data Collection Tools and Quality Assurance

Data were collected using a structured questionnaire which had questions of sociodemographic and economic variables, basic knowledge, attitude and stigmatization on TB, accessibility and availability of TB services, and delay questions. The questionnaire was prepared in English and subsequently translated to the Nepali language. In addition, validity of the questionnaire was checked by three experts. Field activities were conducted to ensure minimum inconvenience to subjects and maximum cooperation was achieved. This involved a trade-off between the need for certain activities and the convenience of the participants. It was important to consider the timing, the place, and the frequency of the activities. The participants were not interrupted unnecessarily in their daily work. The working hours of the enumerators therefore were tailored to the activities of the study subjects and not the other way around. In addition, this study required additional interviewers apart from the researcher to collect the data. The interviewers were paramedics (health assistants) who had basic training on medicine and surgery for three years after finishing ten years of schooling. They received an interview guideline and formal training before the commencement of the survey.

### 2.5. Definition of Patient Delay

Patient delay is the time interval from the appearance of the first symptoms of tuberculosis until the first visit to any formal health care facility (health centers, hospitals, or DOTS centers) [9, 12, 13, 20–27]. Symptom onset is referred to the time at which the first symptom (i.e., persistent cough, fever, weakness, and weight loss or chest pain) of the illness in which a patient seeks care began [11, 15]. Most of the study has been dichotomized >30 days as a prolonged delay [11, 28–30], but some studies have considered a >3-week delay as an unacceptable patient delay [20]. In this study, a period ≥30 days was considered as an unacceptable patient delay.

### 2.6. Statistical Analysis

Data were entered in Epi-Data (Version 3.1) and transferred to STATA (Version 13, Stata Corporation, College Station TX) for analysis. The categorical data were reported as number and percentage. Mean, standard deviation, median, and range (minimum : maximum) were described for continuous variables. The proportion of subjects with ≥30-day delay was estimated. Odds ratios (OR) and their 95% confidence intervals (CI) were estimated using unconditional logistic regression with delay as an outcome. Bivariate analysis was performed to measure the effect of each variable of interest on risk factors of prolonging delay. Regarding the scoring technique that we adopted for this study, the first and foremost step that we took was to reverse the scores before adding them to their domain. It was done so to reflect the increment in the variables that we studied. Moving beyond, we calculated the percentage score for knowledge using the following technique: (Sum of scores obtained/maximum possible score that could be obtained) × 100 [31]. Percentiles scores were computed following the previous step and the studied variables were expressed as lying within a range of 0% to 100%, with the highest percentage reflecting the increase in the characteristic/variable. In addition, scores exceeding 80% were considered as good knowledge of TB. Similarly, to assess the attitude level and stigmatization, we administrated eight and seven questions, respectively. Each question had score of 1–5 (strongly disagree–strongly agree), and a score of >60% was considered to be either a good attitude or an experience of stigma.

Multivariate analysis was performed by multiple logistic regression including variables that showed a significant statistical effect in prediction of prolonging delay in bivariate analysis. Variables associated with delay in the bivariate analysis (*p* ≤ 0.25) were included in the model. Statistical significance was taken as *p* < 0.05. These estimations incorporated treatment centers as a cluster variable so that the standard error can be correctly estimated. Logistic regression implemented under the generalized linear model (GLM) was used to control the clustering effect at both bivariate and multivariate analysis.

## 3. Results

A total of 374 new pulmonary TB patients were interviewed. The median age was 35.03 years. 234 (62.57%) patients were males, 292 (78.07%) were Hindus, and 33.42% were residing in rural areas. As many as 32.89% of the respondents did not finish primary formal education (Table 1). One-fourth of patients were farmers and 26.74% of the respondents were unemployed. The median monthly income was USD 170; however, the lowest earned was only USD 10.

Cough was the most common symptom noticed before diagnosis (69.52%). Less than a quarter (21.56%) of the patients lived more than 5 km away from the facility where TB was diagnosed. More than half (52.67%) of the patients first sought care from a public health facility. Nonpublic health facilities were visited by 159 (42.51%), whereas 4.81% of the patients visited a traditional healer for their first consultation. Only 78 (20.86%) of the respondents consulted chest specialists.

### 3.1. Patient Delay and Determinants

The median patient delay was 32 (IQR 11–70) days. There was unacceptable patient delay in 199 respondents (53.21%). In the bivariate analysis it was found that the factors independently associated with unacceptable delay included the following (Table 2): being Buddhist (OR = 1.46, 95% CI: 1.17–1.83) and residing in the rural areas of Nepal (OR = 2.15, 95% CI: 1.39–3.33). Chest pain was the only first symptom that showed an association with the unacceptable patient delay (OR = 1.5, 95% CI: 1.04–2.17). In addition, living more than 5 km far from the DOTS center (OR = 2.64, 95% CI: 1.58–4.39), being diagnosed and treated by medical officer (OR = 1.72, 95% CI: 1.24–2.37), and consulting chest specialist (OR = 2.98, 95% CI: 1.44–6.13) were also significantly associated with unacceptable patient delay.

The multivariate analysis shows that (Table 3) those who live in rural areas had 3.10 times higher chance to have an unacceptable patient delay than those who were living in urban areas (adj. OR = 3.10, 95% CI: 1.10–8.72). In addition, unemployment was significantly influencing patient delay (adj. OR = 7.79, 95% CI: 1.64–37.00). Distance to reach a DOTS center was strongly associated with patient delay with adj. OR = 5.53, 95% CI: 2.18–13.99. On the other hand, being Muslim was a protective factor for unacceptable delay (adj. OR = 0.10, 95% CI: 0.03–0.77), and those who have smoked more than 5 cigarettes per day (adj. OR = 0.22, 95% CI: 0.08–0.65) were less likely to have an unacceptable patient delay when controlled for other covariates.

## 4. Discussion

This is the first study in central Nepal that assessed the prevalence and determinants of patient delay. Nepal is a low income country and a number of studies have revealed that patient delay varies in countries with relatively lower and middle income. In addition, most studies have revealed that patients often do not clearly notice the symptoms at the onset of the disease, which is one of the reasons for delay [11].

Our study found that the unacceptable patient delay of ≥30 days was 53.21% (95% CI: 48.12–58.28%) which was slightly lower than a study of Nepal in 2009, which showed that 73% of the study population faced patient delay [29]. The differences could be due to the variation in the definition of patient delay and selection of study population. Our study defined the patient delay as the time interval from the appearance of the first symptoms of tuberculosis until the first visit to “any formal health care facility,” whereas some studies defined it as “any health facility.” On the other hand, this study was only focused on sputum positive pulmonary TB patients, whereas the previous study of Nepal included sputum negative and extrapulmonary TB cases. In addition, the DOTS centers have also been expanded, and simultaneously the educational status of the people improved within these years. Therefore, this study has a lower prevalence of patient delay than that of the previous study. However, the result is similar to the study conducted in Uganda [20, 22] and Ethiopia. Higher unacceptable patient delay has been seen in Tanzania, that is, 90% [32].

We found that the patient delay was significantly associated with those who believed in the Buddhist religion and the people living in the rural areas of Nepal (OR = 2.15, 95% CI: 1.39–3.33). This may be because the people with Buddhist religion were more likely to be living in the rural areas where the availability of the health facilities is very poor. Multiple studies have shown that the place of residence leads to the patient delay [21], including a WHO study [31] conducted in seven developing countries and a study conducted in Nigeria. In addition, the result from multivariate analysis showed that being Muslim was protective factor for unacceptable patient delay. This may be because the Muslim populations are concentrated in the terai region which has flat topography and the accessibility to the treatment center is far better than that of mountain and hilly regions.

Moreover, this study observed that the unemployment status of patients leads to an unacceptable delay. It has been suggested that the increased delay observed among farmers or people who do not have paid employment may be related to their socioeconomic condition, specifically lower education, and high poverty [21]. In Nepal, one-fourth of the population is still under poverty line [4, 6].

Furthermore, gender, age, and marital status were not significantly associated with patient delay even though some studies have shown that females were more likely to be associated with delay. Some found that the age group > 45 years [21, 22] can significantly prolong delay. Symptoms like chest pain were contributing factors for prolonging delay. This is due to the lack of knowledge regarding tuberculosis and its symptoms. People generally thought that cough and having fever in the evening are the only symptoms of TB. Thus, they ignore the chest pain and face delay.

Another factor associated with prolonged patient delay was the distance between a DOTS center and their residence (adj. OR = 5.53, 95% CI: 2.18–13.99). This may be due to the geographical condition of Nepal. Health facilities are also not sophisticated enough to diagnose and are not convenient in all rural areas. These situations are similar to the findings of a study conducted in Ethiopia [22]. The medical officers and chest specialist who diagnosed the respondents had significantly increased delay, because almost all specialized services are centralized in Nepal. However, patients who smoked more than 5 cigarettes per day significantly reduced unacceptable patient delay. This may indicate that patients may think that they are at high risk from smoking and that their cough might develop into tuberculosis. Therefore, they were more likely to seek medical services as soon as possible, resulting in smoking being a protective factor for unacceptable patient delay.

## 5. Strength and Limitation of the Study

This study is the first of its kind with a high number of representative samples to be conducted in the study area. Despite the severe geographical challenges posed by different ecological regions, this study has overcome obstacles and covered all three ecozones of the central development region of Nepal. In addition, this study has solely focused on new sputum positive pulmonary tuberculosis patients.

As the study collected the historical data regarding the symptoms of the disease and the first consultation with health facilities, the study might be prone to recall bias.

## 6. Conclusions

Duration of delay for the first consultation was significantly associated with the patient's occupation, income, and persistence of symptoms. The place of residence, distance of the health center from home, and the center of first contact for diagnosis indicate a lack of availability and accessibility of health services in the central development region, even though the studied region is more developed than other regions of Nepal. Therefore, expansion of the DOTS services and increasing the awareness on TB with assured quality will reduce the burden.

## Figures and Tables

**Table 1:** Baseline characteristics of participants.

Characteristics	Number (%)
*Gender*	
Male	234 (62.57)
Female	140 (37.43)
*Age (years)*	
15–29	149 (39.84)
30–44	85 (22.73)
≥45	140 (37.43)
Mean (SD)	39.68 (18.6)
Median (min : max)	35.03 (15.07 : 100.56)
*Religion*	
Hindu	292 (78.07)
Buddha	60 (16.04)
Muslim	22 (5.88)
*Residence*	
Urban	249 (66.58)
Rural	125 (33.42)
*Education*	
Illiterate/read & write	123 (32.89)
Primary	69 (18.45)
Secondary	107 (28.61)
University	75 (20.05)
*Occupation*	
Agriculture	93 (24.87)
Housewife	42 (11.23)
Service	50 (13.37)
Business	58 (15.51)
Unemployed	100 (26.74)
Labour	31 (8.29)
*Income (monthly in USD)*	
<50	35 (11.08)
50–75	24 (7.59)
≥75	257 (81.33)
Mean (SD)	270.87 (467.32)
Median (min : max)	170 (10 : 3800)
*Number of cigarettes per day*	
<5	37 (24.34)
≥5	115 (75.66)
*Symptoms present before diagnosis*	
Cough	260 (69.52)
Fever	56 (14.97)
Loss of weight	17 (4.55)
Hemoptysis	20 (5.35)
Chest pain	21 (5.61)
*Family support*	
None	126 (33.69)
Husband/wife	76 (20.32)
Parents	101 (27.01)
Child	71 (18.98)
*Distance to reach the TB center*	
<5 km	291 (78.44)
≥5 km	80 (21.56)
Mean (SD)	4.25 (11.82)
Median (min : max)	2 (1 : 200)
*Centre of first contact*	
Traditional healer/self-treated	18 (4.81)
Private practitioner/pharmacist/vendor	159 (42.51)
Government health facilities	197 (52.67)
*Categories of HCP for TB diagnosis*	
Paramedics	87 (23.26)
Medical officer	209 (55.88)
Chest specialist	78 (20.86)
*Patient delay (in days)*	199 (53.21)
No delay (<30 days)	175 (46.79)
Delay (≥30 days)	199 (53.21)
Mean (SD)	78.64 (233.94)
Median (IQR)	32 (11–70)

**Table 2:** Factors associated with patient delay (PD): bivariate analysis.

Factors	Number (% PD)	Crude OR	95% CI	*p* value
*Gender*				0.511
Male	234 (53.85)	1		
Female	140 (52.14)	0.93	0.76 to 1.15	
*Age (years)*				0.759
15–29	149 (51.01)	1		
30–44	85 (51.76)	1.03	0.75 to 1.43	
≥45	140 (56.43)	1.24	0.68 to 2.28	
*Religion*				<0.001
Hindu	292 (52.40)	1		
Buddha	60 (61.67)	1.46	1.17 to 1.83	
Muslim	22 (40.91)	0.63	0.38 to 1.03	
*Residence*				<0.001
Urban	249 (46.99)	1		
Rural	125 (65.60)	2.15	1.39 to 3.33	
*Education*				<0.001
Illiterate/read & write	123 (62.60)	1		
Primary	69 (57.97)	0.82	0.59 to 1.16	
Secondary	107 (42.06)	0.43	0.32 to 0.59	
University	75 (49.33)	0.58	0.29 to 1.16	
*Occupation*				<0.001
Agriculture	93 (65.59)	1		
Housewife	42 (47.62)	0.47	0.32 to 0.71	
Service	50 (44.00)	0.41	0.15 to 1.13	
Business	58 (48.28)	0.49	0.15 to 1.56	
Unemployed	100 (52.00)	0.56	0.41 to 0.79	
Labour	31 (51.61)	0.56	0.29 to 1.08	
*Income (monthly in NRs)*				0.009
<5000	35 (68.57)	1		
5000–7500	24 (58.33)	0.64	0.29 to 1.43	
≥7500	257 (50.58)	0.47	0.27 to 0.79	
*Number of cigarettes per day*				0.063
<5	37 (70.27)	1		
≥5	115 (53.91)	0.49	0.24 to 1.04	
*Symptoms present before diagnosis*				<0.001
Cough	260 (51.92)	1		
Fever	56 (57.14)	1.23	0.67 to 2.27	
Loss of weight	17 (52.94)	1.04	0.26 to 4.12	
Hemoptysis	20 (50.00)	0.92	0.61 to 1.41	
Chest pain	21 (61.90)	1.50	1.04 to 2.17	
*Family support*				0.001
None	126 (55.56)	1		
Husband/wife	76 (53.95)	0.94	0.48 to 1.82	
Parents	101 (42.57)	0.53	0.29 to 1.17	
Child	71 (63.38)	1.38	0.56 to 3.41	
*Distance to reach the TB center*				<0.001
<5 km	291 (48.45)	1		
≥5 km	80 (71.25)	2.64	1.58 to 4.39	
*Centre of first contact*				<0.001
Traditional healer/self-treated	18 (72.22)	1		
Private practitioner/pharmacist/vendor	159 (49.69)	0.38	0.10 to 1.39	
Government health facilities	197 (54.31)	0.46	0.13 to 1.64	
*Categories of HCP for TB diagnosis*				0.002
Paramedics	87 (40.23)	1		
Medical officer	209 (53.59)	1.72	1.24 to 2.37	
Chest specialist	78 (66.67)	2.98	1.44 to 6.13	

**Table 3:** Factors associated with patient delay: multivariate analysis.

Factors	Number (% PD)	Crude OR	Adj. OR	95% CI	*p* value
*Religion*					<0.001
Hindu	292 (52.40)	1	1		
Buddha	60 (61.67)	1.46	1.86	0.91 to 3.79	
Muslim	22 (40.91)	0.63	0.10	0.03 to 0.77	
*Residence*					0.032
Urban	249 (46.99)	1	1		
Rural	125 (65.60)	2.15	3.10	1.10 to 8.72	
*Education*					0.135
Illiterate/read & write	123 (62.60)	1	1		
Primary	69 (57.97)	0.82	0.89	0.21 to 3.76	
Secondary	107 (42.06)	0.43	0.46	0.06 to 3.79	
University	75 (49.33)	0.58	0.96	0.08 to 11.19	
*Occupation*					<0.001
Agriculture	93 (65.59)	1	1		
Housewife	42 (47.62)	0.47	1.65	0.18 to 14.97	
Service	50 (44.00)	0.41	3.89	0.35 to 43.32	
Business	58 (48.28)	0.49	1.79	0.05 to 70.94	
Unemployed	100 (52.00)	0.56	7.79	1.64 to 37.00	
Labour	31 (51.61)	0.56	0.830	0.17 to 4.02	
*Income (monthly in NRs)*					0.116
<5000	35 (68.57)	1	1		
5000–7500	24 (58.33)	0.64	13.34	0.35 to 511.0	
≥7500	257 (50.58)	0.47	0.94	0.04 to 21.41	
*Number of cigarettes in a day*					0.006
<5	37 (70.27)	1	1		
≥5	115 (53.91)	0.49	0.22	0.08 to 0.65	
*Symptoms present before diagnosis*					0. 573
Cough	260 (51.92)	1	1		
Fever	56 (57.14)	1.23	1.56	0.58 to 4.20	
Loss of weight	17 (52.94)	1.04	1.24	0.36 to 4.22	
Hemoptysis	20 (50.00)	0.92	0.05	0.06 to 4.14	
Chest pain	21 (61.90)	1.50	3.58	0.13 to 95.89	
*Family support*					<0.001
None	126 (55.56)	1	1		
Husband/wife	76 (53.95)	0.94	1.19	0.22 to 8.65	
Parents	101 (42.57)	0.53	0.66	0.11 to 2.47	
Child	71 (63.38)	1.38	4.12	0.61 to 27.98	
*Distance to reach the TB center*					<0.001
<5 km	291 (48.45)	1	1		
≥5 km	80 (71.25)	2.64	5.53	2.18 to 13.99	
*Centre of first contact*					0.443
Traditional healer/self-treated	18 (72.22)	1	1		
Private practitioner/pharmacist/vendor	159 (49.69)	0.38	0.50	0.12 to 2.09	
Government health facilities	197 (54.31)	0.46	0.97	0.12 to 7.86	
*Categories of HCP for TB diagnosis*					0.929
Paramedics	87 (40.23)	1	1		
Medical officer	209 (53.59)	1.72	1.29	0.34 to 4.98	
Chest specialist	78 (66.67)	2.98	0.99	0.29 to 3.43	
